# Hierarchical virtual screening for the discovery of new molecular scaffolds in antibacterial hit identification

**DOI:** 10.1098/rsif.2012.0569

**Published:** 2012-08-29

**Authors:** Pedro J. Ballester, Martina Mangold, Nigel I. Howard, Richard L. Marchese Robinson, Chris Abell, Jochen Blumberger, John B. O. Mitchell

**Affiliations:** 1 European Bioinformatics Institute, Wellcome Trust Genome Campus, Hinxton, Cambridge CB10 1SD, UK; 2 Department of Chemistry, University of Cambridge, Lensfield Road, Cambridge CB2 1EW, UK; 3 Department of Physics and Astronomy, University College London, London WC1E 6BT, UK; 4 Biomedical Sciences Research Complex and EaStCHEM School of Chemistry, University of St Andrews, North Haugh, St Andrews, Fife KY16 9ST, UK

**Keywords:** virtual screening, antibacterial hit identification, chemoinformatics, bioinformatics, machine learning, high-throughput screening

## Abstract

One of the initial steps of modern drug discovery is the identification of small organic molecules able to inhibit a target macromolecule of therapeutic interest. A small proportion of these hits are further developed into lead compounds, which in turn may ultimately lead to a marketed drug. A commonly used screening protocol used for this task is high-throughput screening (HTS). However, the performance of HTS against antibacterial targets has generally been unsatisfactory, with high costs and low rates of hit identification. Here, we present a novel computational methodology that is able to identify a high proportion of structurally diverse inhibitors by searching unusually large molecular databases in a time-, cost- and resource-efficient manner. This virtual screening methodology was tested prospectively on two versions of an antibacterial target (type II dehydroquinase from *Mycobacterium tuberculosis* and *Streptomyces coelicolor*), for which HTS has not provided satisfactory results and consequently practically all known inhibitors are derivatives of the same core scaffold. Overall, our protocols identified 100 new inhibitors, with calculated *K*_i_ ranging from 4 to 250 μM (confirmed hit rates are 60% and 62% against each version of the target). Most importantly, over 50 new active molecular scaffolds were discovered that underscore the benefits that a wide application of prospectively validated *in silico* screening tools is likely to bring to antibacterial hit identification.

## 1. Introduction

In recent years, bacterial epidemics have been fuelled by the emergence of multi-drug-resistant pathogen strains, which increasingly challenge existing treatments [1]. Despite this growing threat, many new antibiotic candidates are chemical molecules re-engineered from old drug classes discovered decades ago for which there are already underlying resistance mechanisms [1–4]. Fischbach & Walsh [5] have argued that, while making incremental improvements to existing scaffolds is a good short-term strategy for refilling the antibiotic pipeline, the discovery of new molecular scaffolds should be a priority owing to the emergence of multi-drug-resistance among pathogens and the need for a sustainable plan for combating resistance. Simmons *et al*. [6] go even further by pointing out that the discovery of drugs with novel modes of action will be vital to meet the threats created by the emergence of resistance [7]. With the deciphering of the genome sequences of major human pathogens [8], many companies vigorously pursued the identification of novel antibiotic agents from high-throughput screening (HTS) campaigns using purified enzyme targets that were validated by genomic approaches as being essential for the viability of the pathogen [6]. The expected outcome was the production of brand new inhibitor classes against these novel targets that could eventually lead to innovative drugs and hence reduced likelihood of resistance emerging rapidly. Furthermore, finding a high number of inhibitor classes was an important requirement, as multiple high-quality hits are needed to counteract the attrition in the subsequent hit-to-lead, lead optimization and clinical stages required to generate a novel antibiotic [3].

Unfortunately, the performance of HTS against post-genomic antibacterial targets has generally been unsatisfactory. Payne *et al*. [3] critically assessed the results of what these authors describe as an unprecedented concentration of screening resource for a single therapy area. From the 70 antibacterial HTS campaigns run between 1995 and 2001 (67 target-based and three whole cell), it was found that a mere 16 HTS gave rise to hits and only five of these ultimately resulted in leads (i.e. molecules that not only inhibited the enzyme target, but also reduced the growth of the pathogen *in vitro*). On the basis of GlaxoSmithKline (GSK) metrics, the success rate from antibacterial HTS was four- to fivefold lower than for targets from other therapeutic areas available at that time. These authors concluded that this was a disappointing and financially unsustainable outcome, especially in view of the length of time devoted to these experiments and considering that the costs per HTS campaign were estimated then to be around US$1 million. Payne *et al*. [3] also argued that the difficulty of finding antibacterial hits from HTS was not unique to GSK, a view further supported by Simmons *et al*. [6], who consider that success in discovering inhibitors, using HTS of chemical libraries is rare in this area.

The poor efficacy of HTS in this area has been attributed to the limited chemical diversity of the screened collections [3] and to the assumption that antibacterial targets have intrinsically low druggability [9]. Remarkably, well-known technical difficulties such as false negatives in HTS have not been investigated as a contributor to poor efficacy despite false negatives probably occurring frequently [10], representing typically an estimated 15–26% of the total number of actives [11] and having a particularly large impact on hard targets [12]. Most importantly, the cost and slow operation of HTS imposes the selection of a relatively small set of promising compounds that in an extreme case may not contain inhibitors. Hence, while the false-positive rate of HTS will generally be much better than that from a virtual screening technique applied to the same compound library, the fact that virtual screening can quickly search a much larger portion of the chemical space means that the latter has, in principle, access to many more inhibitors and consequently may find more inhibitors in some cases. As a result of this situation, lead discovery has generally become a key bottleneck for the development of new treatments for infectious diseases [13].

A consensus is now emerging that new approaches for finding antibacterial inhibitors are required. Fischbach & Walsh [5] consider that retooled target-based strategies can play an important role in lead discovery. Hopkins *et al.* [9] find that new, more cost-effective and efficient methods of drug discovery are urgently required if we are to tackle the multiple global health challenges of emerging and neglected infectious diseases for which there is relatively little basic science investment. Recently, Simmons *et al*. [6] have made a compelling case for the use of structure-based virtual screening for antibacterial hit identification. Indeed, the application of advanced computational methods to predict molecular bioactivity has distinctive advantages such as much reduced time scales and financial costs that enable the effective exploration of extremely large molecular databases against a high number of validated drug targets.

In this study, we investigate a novel computational methodology that exploits the structures of antibacterial targets in order to identify brand new classes of inhibitors. We combine state-of-the-art docking protocols hierarchically with ultrafast shape recognition (USR) capable of quickly identifying database molecules that are similarly shaped to a known inhibitor. In this way, only the molecules that can fit the target's active site, and hence are likely to bind to the enzyme, are subsequently fed to the much more computationally demanding docking calculation. This hybrid approach permits the effective exploration of truly large and diverse molecular databases in a time- and resource-efficient manner while exploiting both ligand and protein structure data, as a way to increase the likelihood of identifying new active scaffolds that could be unaffected by existing resistance mechanisms. The methodology is tested prospectively on two versions of a post-genomic antibacterial target (type II dehydroquinase from *Mycobacterium tuberculosis* and *Streptomyces coelicolor*), which is representative of this situation in that HTS has not provided satisfactory results and consequently little scaffold diversity is currently known. Testing any resulting hit for whole-cell antibacterial activity would be necessary to determine which of these enzyme inhibitors are able to reach the intracellular target with sufficient concentration, which implies crossing the bacterial membrane and being unaffected by resistance mechanisms such as efflux pumps. Such an extensive follow-up work is however out of the scope of this study.

## 2. Results and discussion

**Figure 1.**
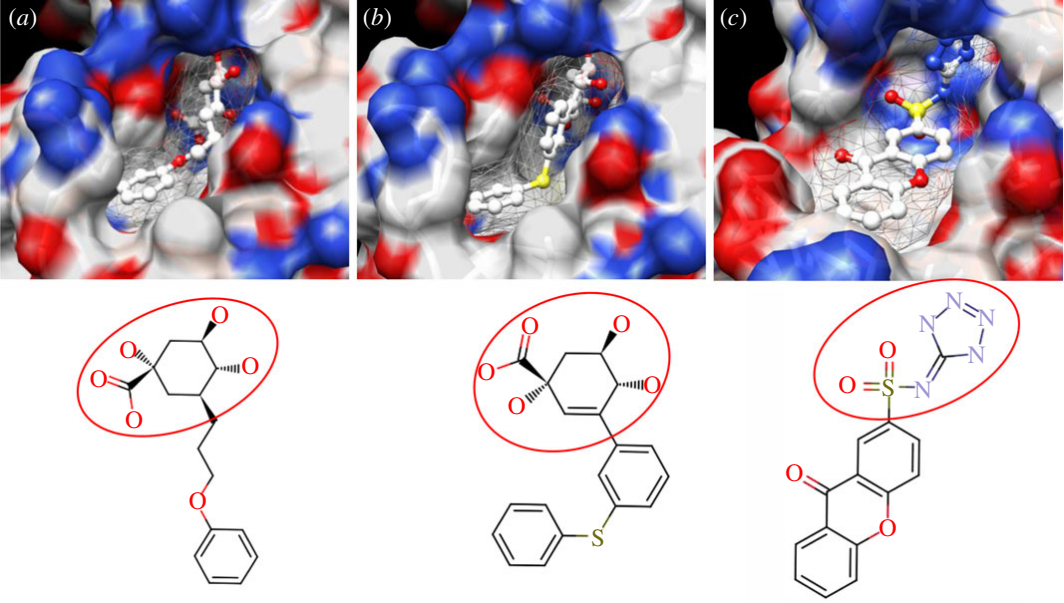
Visualization of the three co-crystallized ligands used as templates for the shape similarity screen ((*a*) CA2 complexed with *S. coelicolor* DHQase; (*b*) RP4 complexed with *S. coelicolor* DHQase; (*c*) GAJ complexed with *H. pylori* DHQase). The van der Waals surface of each bound molecule is represented as a grid to show the high degree of shape complementarity between the ligands and their receptors. The core scaffold, defined as that closest to the catalytic residues, is circled. CA2 and RP4 are derivatives of the transition state structure (core scaffold 2,3-anhydroquinic acid which is also the crystallographic ligand FA1), whereas the innovative structure of GAJ was identified with HTS [18].

The enzyme 3-dehydroquinate dehydratase (dehydroquinase; EC 4.2.1.10) catalyses the reversible dehydration of 3-dehydroquinate to form 3-dehydroshikimate [14–16]. Type II dehydroquinase (DHQase for short) is the third enzyme of the Shikimate pathway, which is essential for the viability of bacteria such as *M. tuberculosis*, *S. coelicolor* and *Helicobacter pylori* [17]. This pathway is present in bacteria, fungi, plants and apicomplexan parasites, but not in mammals, and hence represents an ideal target for the development of antibacterial agents, as these agents would be expected to have a spectrum of antibacterial activity restricted to those human pathogens expressing DHQase such as *M. tuberculosis* and *H. pylori*. An HTS of some 150 000 compounds against *H. pylori* DHQase was used as a starting point to identify novel inhibitors [18]. While approximately 100 molecules with more than 50 per cent inhibition of *H. pylori* DHQase enzyme activity at a concentration of 20 μg ml^−1^ were identified in the primary screening, only one confirmed inhibitor against *H. pylori* DHQase was reported (the ligand named GAJ in figure 1, which inhibited this enzyme with *K*_i_ = 20 μM). This molecule also showed micromolar activity against *S. coelicolor*^1^ DHQase (*K*_i_ = 230 μM) but only residual activity against the *M. tuberculosis* enzyme (10% inhibition at 200 μM). The ChEMBL database (https://www.ebi.ac.uk/chembl/ last accessed on 31 January 2012), which has been estimated [9] to contain 90 per cent of the published medicinal chemistry structure–activity data, shows that practically all existing DHQase inhibitors are derivatives of the same core scaffold (2,3-anhydroquinic acid or anhydroquinate ring, the reaction intermediate), consistent with the successful use of rational drug design approaches and the typically low performance of HTS on antibacterial targets. Figure 1 shows the chemical structures of these active scaffolds as well as the high degree of shape complementarity between these molecules and their respective receptors.

Our search for new classes of DHQase inhibitors was carried out on a molecular database built from the ZINC resource [19]. With almost nine million commercially available molecules, its size is between 17 and 59 times higher than those previously used for large-scale HTS campaigns (from 150 000 to 530 000 compounds [3,18]) and, to the best of our knowledge, the largest that has ever been used in a successful prospective virtual screen. Such a wealth of chemical diversity is a key component of our screen, as a smaller database generated with the same procedure would have contained a lower number of innovative scaffolds. In order to compile a subset of molecules likely to fit the active site, we searched for molecules that are similarly shaped to known inhibitors using USR [20]. USR is an unusually rapid descriptor-based shape similarity technique [21], which is particularly suited for scaffold hopping and has already been successfully applied to the identification of brand new active scaffolds within very large molecular databases [22]. It is well known that using several molecules as search templates results in a broader exploration of different regions of chemical space and thus we ran USR using each of the DHQase ligands shown in figure 1 as templates (CA2 from PDB entry 2BT4, RP4 from 2CJF and GAJ from 2C4W). This process resulted in the identification of 4379 diverse molecules that are similar in shape to these inhibitors, and thus fit the DHQase active site, from the nine million molecules initially considered.

These similarly shaped molecules were thereafter inspected by an *in silico* model intended to remove those predicted to be toxic. Toxicity screens during the early stages of drug discovery should prioritize specificity (the proportion of correctly identified non-toxins) over sensitivity (the proportion of correctly identified toxins), in order to reduce the likelihood of erroneously discarding good hits at a stage when attrition costs are low [23]. Indeed, our aim is to remove obviously toxic molecules rather than performing the exhaustive toxicity analysis that would be required at later stages. Thus, our model removed compounds predicted to be both carcinogenic and mutagenic, which are recognized as serious forms of toxicity in the context of drug discovery [24]. By discarding compounds predicted to exhibit both types of toxicity, we sought to reduce the loss of non-toxins as a result of imperfect predictions of each individual type. This screen removed 471 molecules from the 4379 molecules analysed.

The resulting 3908 molecules were docked into a panel of DHQase protein structures. We selected five ligand-bound DHQase X-ray crystal structures to be used for docking: three structures of the *S. coelicolor* version of the enzyme (PDB codes 1GU1, 2BT4 and 2CJF), one structure for *H. pylori* DHQase (2C4W) and the fifth structure for *M. tuberculosis* DHQase (1H0R). The purpose of considering more than one X-ray structure is twofold. First, because each structure represents at least a slightly different conformation of the protein in the crystal, we will be addressing the flexibility of the protein to some extent. Second, because the structures refer to three closely related versions of the same enzyme coming from three different organisms (two pathogenic bacteria and a commonly used non-pathogenic model organism), these data will enable the search for molecules potentially able to kill all three organisms. As expected, this process resulted in a similar number of docking poses against each target: 21 788 (1GU1), 28 191 (1H0R), 21 771 (2BT4), 24 814 (2C4W) and 20 976 (2CJF). Pose generation quality was investigated by re-docking the three largest co-crystallized ligands (CA2 from 2BT4, RP4 from 2CJF and GAJ from 2C4W) back to their respective receptors, with root-mean-square deviation (r.m.s.d.) values between co-crystallized and re-docked poses being 0.59 (2CJF), 0.79 (2BT4) and 2.28 (2C4W). The r.m.s.d. over 2 Å indicates that there may be significant errors in pose generation, and thus a more exhaustive search should lead to improved performance. In addition to ChemScore [25] values arising from the pose generation process, each of these five sets of docking poses were re-scored with GoldScore [26] and ASP [27]. Protocol 1 is a consensus scoring [28] strategy that considered the three sets of docking poses containing the three largest co-crystallized ligands (CA2 from PDB code 2BT4, RP4 from 2CJF and GAJ from 2C4W), and each set was sorted with the average rank of the pose according to ChemScore, GoldScore and ASP (this consensus score will be henceforth referred to as GOLD-3). High ranking poses by three different scoring functions represent by construction a more reliable prediction than any of the constituent scoring functions alone. In practice, consensus scoring has been generally found to improve virtual screening performance dramatically with respect to the individual scoring functions [28,29].

The RF-Score [30] is a member of a new class of scoring functions that use non-parametric machine learning to build predictive models of binding affinity in an entirely data-driven manner. RF-Score has been rigorously shown [30,31] to perform better than 16 standard scoring functions in ranking protein–ligand complexes according to predicted binding affinity. Protocol 2 used RF-Score alone to re-score and rank the same three sets of docking poses as in protocol 1. The reason for restricting our study to three of the five docking sets was that we wanted to determine the rank of each co-crystallized ligand according to both protocols, and considered that the ligand in 1GU1 and 1H0R (FA1) was too small to have a competitive potency and thus rank high against the predominantly larger docked molecules. Protocol 1 ranked ligands 2nd (GAJ), 5th (RP4) and 19th (CA2) with respect to the 3908 molecules docked against its corresponding co-crystallized protein (2C4W, 2CJF and 2BT4, respectively), whereas protocol 2 ranked these ligands much lower in each list: 2679th (GAJ), 2865th (RP4) and 3878th (GAJ). Interestingly, the binding affinity prediction of these inhibitors by RF-Score is particularly accurate for a scoring function: p*K*_i_^RF-Score^(2BT4) = 6.30 (+1.82 with respect to measured *K*_i_ = 33 μM [32]), p*K*_i_^RF-Score^(2CJF) = 7.35 (+0.20 with respect to measured *K*_i_ = 70 nM [33]) and p*K*_i_^RF-Score^(2C4W) = 6.28 (+1.58 with respect to measured *K*_i_ = 20 μM [18])^2^. In §3, these seemingly conflicting retrospective results will be discussed in the light of the prospective performance of both protocols. Protocol 3 identified all molecules that ranked in the top 500 against all five targets to encourage the discovery of broad-spectrum inhibitors. Lastly, protocol 4 simply consisted of searching for the most similar molecules to the RP4 ligand using USR, so as to investigate the advantages of additionally exploiting protein structure as in protocols 1–3.

The next step was to purchase the compounds highlighted by these protocols to test them *in vitro* against *M. tuberculosis* DHQase and *S. coelicolor* DHQase. With the modest budget assigned to this proof of concept (£5000), we could purchase 148 compounds (full details on this process can be found in electronic supplementary material, Materials and methods). Tables 1 and 2 show the performance of each virtual screening protocol against each target (all IC_50_ measurements are included in the electronic supplementary material). Protocol 2 performed better than protocol 1 and much better than protocol 3, both in terms of hit rate at IC_50_ ≤ 250 μM and median IC_50_. As protocol 3 was the only strategy exploiting the *M. tuberculosis* DHQase structure while using the same consensus scoring as protocol 1, the difference in performance suggests that focusing on the compounds at the top of the ranked list is more important than using their exact crystal structure. Lastly, while protocol 4 did not identify any inhibitor with IC_50_ ≤ 250 μM, it obtained the best performance in terms of hit rate at a higher cut-off (IC_50_ ≤ 500 μM).

**Table 1.** Performance of each virtual screening protocol against *M. tuberculosis* DHQase. For each protocol, the number of tested compounds, confirmed inhibitors with IC_50_ ≤ 250 μM (hit rate between brackets), confirmed inhibitors with IC_50_ ≤ 500 μM (hit rate between brackets) and indication of potency distribution of these discovered inhibitors (L, lowest IC_50_; M, median IC_50_; H, highest IC_50_).

virtual screening protocol	tested	IC_50_ ≤ 250 μM	IC_50_ ≤ 500 μM	(L, M, H) (μM)
1. USR-3>GOLD::ChemScore>GOLD-3	71	20 (28.2%)	40 (56.3%)	(48, 245, 475)
2. USR-3>GOLD::ChemScore>RF-Score	67	25 (37.3%)	38 (56.7%)	(89, 223, 426)
3. USR-3>GOLD::ChemScore>Top500-5	8	2 (25.0%)	5 (62.5%)	(94, 268, 417)
4. USR-RP4	5	0 (0%)	5 (100%)	(320, 354, 409)
overall performance	148	47 (31.8%)	88 (59.5%)	(48, 243, 475)

**Table 2.** Performance of each virtual screening protocol against *S. coelicolor* DHQase. For each protocol, the number of tested compounds, confirmed inhibitors with IC_50_ ≤ 250 μM (hit rate between brackets), confirmed inhibitors with IC_50_ ≤ 500 μM (hit rate between brackets) and indication of potency distribution of these discovered inhibitors (L: lowest IC_50_, M: median IC_50_, H: highest IC_50_).

virtual screening protocol	tested	IC_50_ ≤ 250 μM	IC_50_ ≤ 500 μM	(L, M, H) (μM)
1. USR-3>GOLD::ChemScore>GOLD-3	71	24 (33.8%)	38 (53.5%)	(57, 208, 490)
2. USR-3>GOLD::ChemScore>RF-Score	67	32 (47.8%)	43 (64.2%)	(8, 203, 478)
3. USR-3>GOLD::ChemScore>Top 500-5	8	2 (25.0%)	6 (75.0%)	(177, 321, 496)
4. USR-RP4	5	0 (0%)	4 (80.0%)	(295, 322, 401)
overall performance	148	58 (39.2%)	91 (61.5%)	(8, 215, 496)

**Figure 2.**
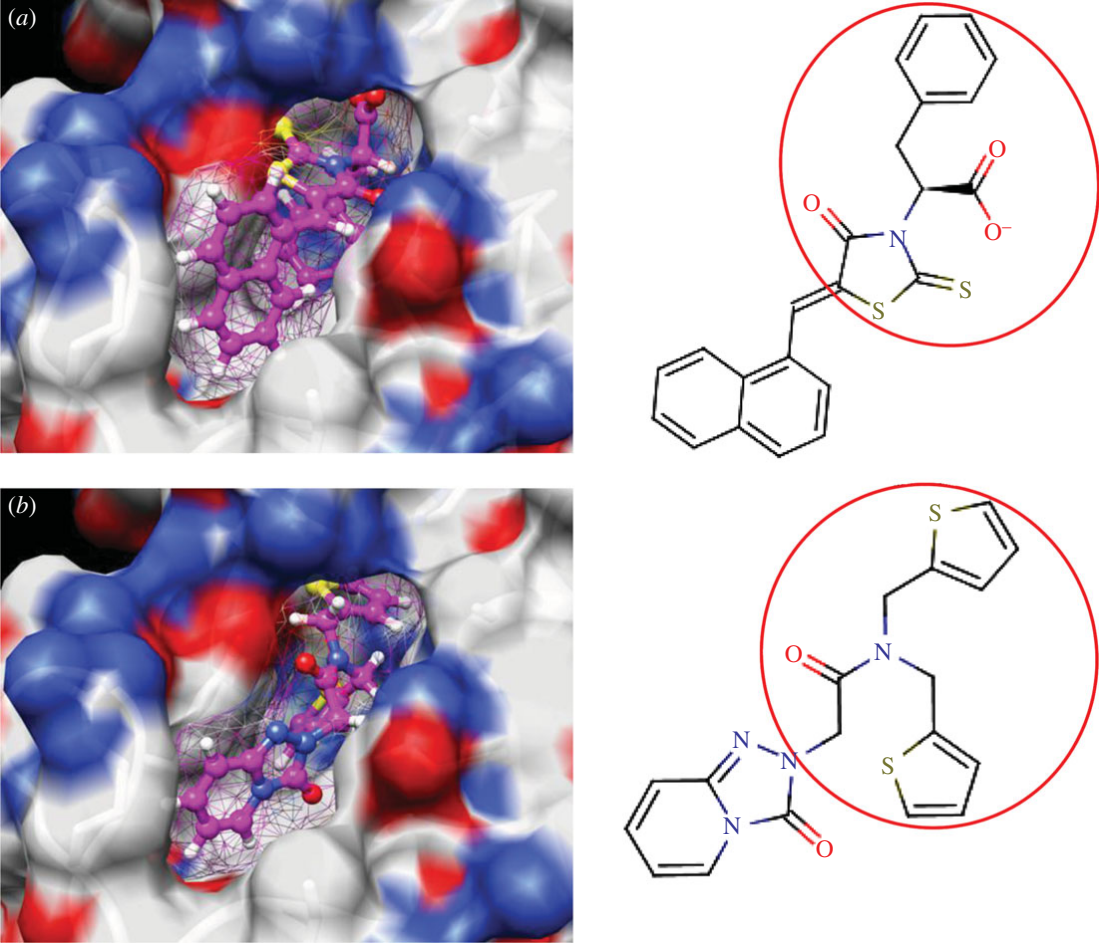
Docking pose and chemical structure of two new inhibitors of *S. coelicolor* DHQase identified with our virtual screening protocols ((*a*) ZINC00978022 with *K*_i_ = 4 μM; (*b*) ZINC24469052 with *K*_i_ = 21 μM). The new core scaffolds, circled in the structure, are strikingly different from those previously known (figure 1).

In order to assess the overall hit rate and potency of these inhibitors, we calculated their inhibition constant (*K*_i_) as explained in electronic supplementary material, Materials and methods (see table 3 for a summary). This is necessary to have a more accurate comparison with hit rates in the literature and with the potency of previously known inhibitors. Our protocols identified 89 inhibitors for *M. tuberculosis* DHQase and 91 inhibitors for *S. coelicolor* DHQase with *K*_i_ ≤ 250 μM (a total of 100 new inhibitors with activity against at least one of the targets). Among the rest of the tested compounds, many showed a small percentage of inhibition at a higher concentration. Overall, hit rates are unusually high, with the confirmed hit rate at the low micromolar range for *S. coelicolor* DHQase (27.0% molecules with *K*_i_ ≤ 100 μM; a total of 40 inhibitors) being noticeably higher than that for *M. tuberculosis* DHQase (23.6%; 35 inhibitors). The trend is still observed when considering a less restrictive activity cut-off (*K*_i_ ≤ 250 μM) and in terms of median potency (114 μM for *M. tuberculosis* versus 108 μM for *S. coelicolor*). This difference might be due to the fact that our protocols are primarily exploiting crystal structures for *S. coelicolor* DHQase. Figure 2 shows examples of these new inhibitors, which are characteristic of the high chemical diversity of the new core scaffolds. Table 4 shows the shape and chemical structure of these five new inhibitors in comparison with the search template used (RP4).

**Table 3.** Overall virtual screening performance in terms of calculated *K*_i_ (148 compounds tested against each version of the enzyme). For each target, confirmed inhibitors with *K*_i_ ≤ 100 μM (hit rate between brackets), confirmed inhibitors with *K*_i_ ≤ 250 μM (hit rate between brackets) and the *K*_i_ of the three most potent inhibitors found (L^1^–L^3^).

overall performance	*K*_i_ ≤ 100 μM	*K*_i_ ≤ 250 μM	(L^1^, L^2^, L^3^) (μM)
against Mtb DHQase	35 (23.6%)	89 (60.1%)	(23, 24, 40)
against Scl DHQase	40 (27.0%)	91 (61.5%)	(4, 21, 29)

**Table 4.** Results for the USR screen of the nine-million single-conformer database using the co-crystallized pose of the highly optimized RP4 as the template, which was the highest ranked molecule as expected. We selected five top-ranking compounds on the basis of their prompt availability from the supplier and tested them *in vitro* against both *M. tuberculosis* DHQase and *S. coelicolor* DHQase. All these molecules showed mid-micromolar activity against both targets, but most importantly new core scaffolds to be used as starting points for optimization were identified. Each core scaffold is circled as predicted by the corresponding docking pose against *S. coelicolor* DHQase (2CJF).

ZINC ID	rank	USR score	shape	two-dimensional structure	*K*_i_ (Mtb; μM)	*K*_i_ (Sc; μM)
RP4	first	1.000	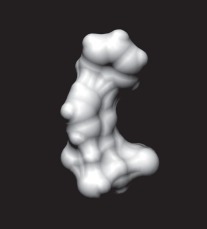	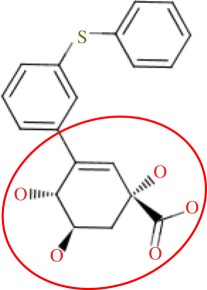	0.38	0.07
ZINC18086350	fifth	0.9404	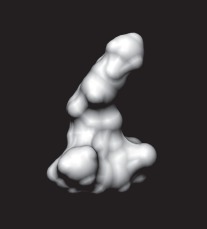	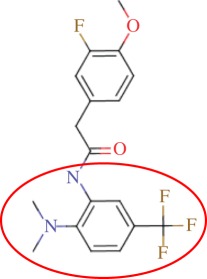	166	45% at 224
ZINC05657290	13th	0.9356	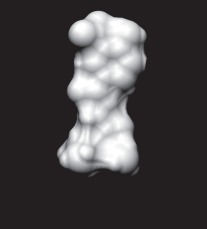	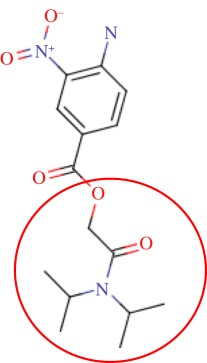	192	201
ZINC06024496	15th	0.9341	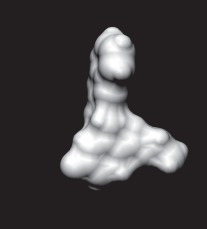	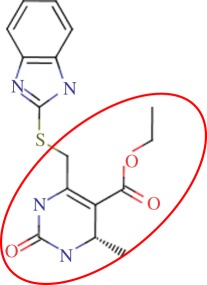	159	174
ZINC15608616	24th	0.9319	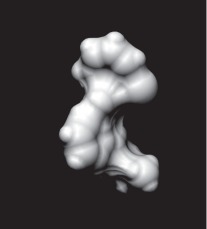	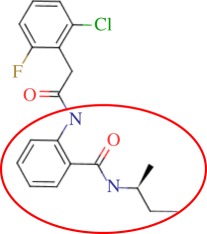	150	149
ZINC10025084	26th	0.9315	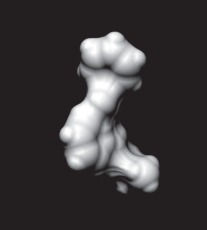	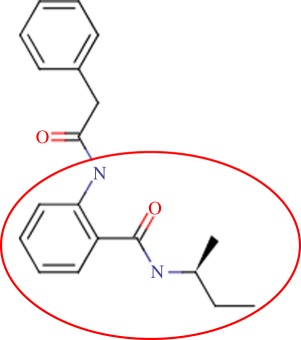	167	148

**Figure 3.**
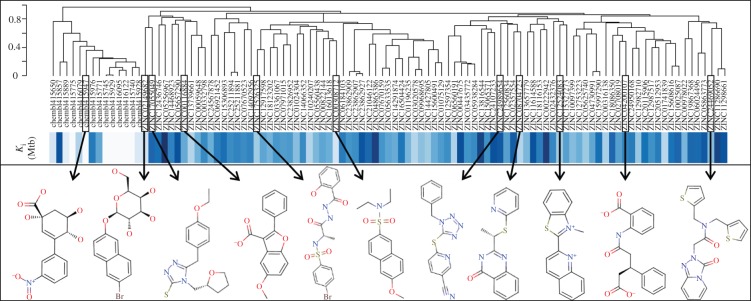
Diversity of new inhibitors of *M. tuberculosis* DHQase. The hierarchical clustering plot at the top includes all the new inhibitors for this version of the target arising from our study (89 molecules identified by their ZINC codes) as well as all the previously known confirmed inhibitors for this target in the ChEMBL database (14 molecules identified by their ChEMBL codes). The dendrogram shows the results of the clustering of these molecules in terms of their chemical structure similarity. The relative *K*_i_ of these molecules is shown as different shades of blue (the darker the shade, the higher the *K*_i_). *K*_i_ values for the 14 ChEMBL inhibitors range from 54 nM to 200 μM (these are mostly optimized hits unlike our new structurally diverse inhibitors, which come directly from virtual screening). Below the *K*_i_ band, representatives from the 48 new core scaffolds are shown. These are strikingly diverse compared to the previously known core scaffold (first cluster on the left).

**Figure 4.**
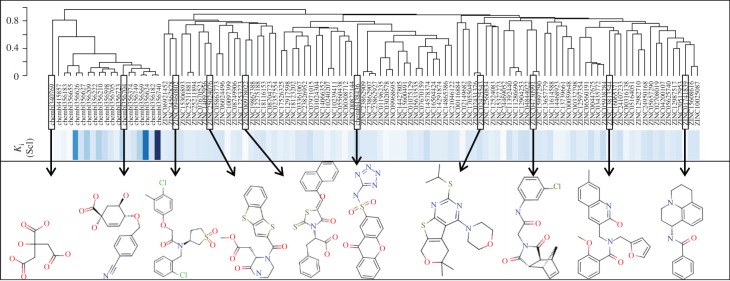
Diversity of new inhibitors of *S. coelicolor* DHQase. The hierarchical clustering plot at the top includes all the new inhibitors for this version of the target arising from our study (91 molecules identified by their ZINC codes) as well as all the previously known confirmed inhibitors for this target in the ChEMBL database (20 molecules identified by their ChEMBL codes). The dendrogram shows the results of the clustering of these molecules in terms of their chemical structure similarity. The relative *K*_i_ of these molecules is shown as different shades of blue (the darker the shade, the higher the *K*_i_). *K*_i_ values for the 20 ChEMBL inhibitors range from 7.3 to 2000 μM (these are mostly optimized hits unlike our new structurally diverse inhibitors that come directly from virtual screening). Five additional chemical series were found for *S. coelicolor* DHQase, which adds to a total of 53 new molecular scaffolds contained in these 100 DHQase inhibitors. These are strikingly diverse compared to most of the previously known core scaffolds (first cluster on the left).

As discussed in §1, the single most important requirement for new inhibitors is having different core scaffolds from those previously known. For our case study, we retrieved all inhibitors with measured *K*_i_ from the ChEMBL database (ChEMBL target IDs 18038 and 20064 for *S. coelicolor* and *M. tuberculosis* DHQase, respectively) and observed that all but two of these ChEMBL inhibitors have FA1 (a transition state analogue shown in figure 1) as the core scaffold. In order to investigate how different our new inhibitors are compared with those previously known, we cluster all these molecules in terms of chemical structure similarity (figures 3 and 4). The clustering dendrogram evidences the striking diversity of new inhibitors. In figure 3 (*M. tuberculosis*), the first cluster at the top groups all the ChEMBL inhibitors that have the same core scaffold. By using the clustering as a preliminary classification, we manually inspected the clusters and found 48 new core scaffolds (all inhibitors sharing the same core scaffold constitute a chemical series by definition). Representatives from these chemical series are pictured on the left of the figure. The results for figure 4 (*S. coelicolor*) are very similar except that one of the ChEMBL inhibitors has a very different chemical structure (chembl438436, which is actually GAJ; the HTS hit in figure 1) and thus appears separated from the FA1 derivatives that are clustered at the top. Five additional chemical series were found for this target, which adds to a total of 53 new molecular scaffolds contained in these 100 DHQase inhibitors.

## 3. Discussion and conclusions

Four virtual screening protocols have been used in this prospective study. This approach was intended to investigate the relative performance of ligand-based and structure-based techniques on the same target and screening database (these parallel prospective applications constitute the most rigorous type of virtual screening method validation, but have not been carried out until now, as discussed in a recent comprehensive survey [34]). Protocols 1 and 2 differ only in that each uses a different re-scoring function to rank docking poses according to predicted strength of binding (GOLD-3 and RF-Score, respectively). Unlike protocol 2, protocol 1 was able to rank the three crystal poses very highly. On the basis of these retrospective results, one might conclude that GOLD-3 is a much more appropriate re-scoring procedure for this target. However, prospectively, RF-Score led to the identification of an outstanding proportion of new inhibitors, and performed even better than GOLD-3 both in terms of hit rate and median potency. Interestingly, RF-Score was ranking different, previously unknown inhibitors higher than the known inhibitors, with no overlap with the inhibitors identified by GOLD-3. This is a reminder of an important limitation of retrospective studies: many of the highly ranked assumed inactives used as decoys could actually be active (especially if their potency is at the level typical of hit identification, not that of optimized leads or even drugs, and decoys are chosen to have physico-chemical properties similar to those of known actives).

Of course, not all the performance can be attributed to the re-scoring functions, as the initial shape similarity screen was used to enrich the few thousand docked molecules with actives. For instance, GOLD-3 ranks the low nanomolar RP4 ligand fifth of the 3908 similarly shaped molecules. However, a simple arithmetic rule of three tells us that this molecule would be expected to be ranked 11 239th if GOLD::ChemScore > GOLD-3 had been applied directly to the entire database of 8 784 580 molecules. As pointed out by Schneider [35], the particularly successful application of USR alone to prospective virtual screening in the past [22] underscores the utility of coarse-grained models for first-pass compound selection. The present application constitutes another example. In order to investigate what exactly was gained by exploiting structural information via docking, we look at the results of protocol 4 in table 4 (tested hits from the USR-RP4 screen). Compared with protocols 1 and 2, which perform docking on the subset preselected by USR (tables 1 and 2), protocol 4 (USR alone) obtained a significantly higher hit rate at the cost of providing a much higher median *K*_i_ (lower potency). These results show that USR leads to a smaller proportion of false positives, which suggests that shape similarity alone is more reliable than pose generation and scoring in docking, at least for this target. By contrast, the addition of estimated binding affinity makes the true positives from docking more potent than those from USR alone. Motivated by these results and just like in lead optimization [36], we propose hit identification to be regarded as a multi-objective problem where screening outcomes (potency, hit rate, chemical diversity, speed and cost of operation) are optimized by searching for the best protocol (methods, templates, structures, database composition and size). This will help us to establish which virtual screening techniques are more appropriate for the requirements of a particular hit identification project.

Beyond methodological findings, the most important contribution of this study is the outstanding diversity in new molecular scaffolds found (a total of 53 new chemical series at a nominal cost of £5000). This is particularly valuable, taking into account that, with the exception of GAJ and ChEMBL340769 (*S. coelicolor*), all previously known inhibitors are derivatives of the transition state. Overall, we have found 100 new DHQase inhibitors comprising a total of 180 bioactivity endpoints against both targets. These results contrast with those from an HTS of 150 000 compounds against the *H. pylori* version of the enzyme, which reported a single confirmed inhibitor with *K*_i_ = 20 μM [18]. In calculating hit rates, we have adopted the same minimum definition of a hit as in industrial HTS campaigns for antibacterial hit identification^3^. As noticed in a comprehensive survey of prospective virtual screening applications [34], low- and mid-micromolar binders against novel targets are commonly reported in leading journals if there is limited ligand information available. This is because after discovery of a new scaffold, its derivatives can be tested to improve potency for that target. Although this task can be challenging, large potency improvements have been achieved in antibacterial targets such as *M. tuberculosis* adenosine 5′-phosphosulfate reductase [37] (10-fold) and *S. coelicolor* DHQase [33] (3000-fold). The latter figure suggest that in principle, a DHQase hit with *K*_i_ = 100 μM (the upper threshold of our definition for low micromolar) could potentially reach 30 nM once optimized.

Several factors have contributed to this level of performance. First, the use of ligand-based shape similarity to enrich the docking library with likely binders has been shown to be instrumental. Three USR searches, each using a different instance of shape complementarity as template, account to some extent for partial shape complementarity between ligand and its receptor as well as shape variation in the binding site due to induced fit effects. Second, while the notion that a certain degree of shape complementarity is necessary for binding has been recognized and implemented in drug design tools for decades, it is only now that the efficiency, effectiveness and widespread availability of such tools are making a large impact in hit identification [22,38–40]. Third, the exploration of such a large molecular database, effortlessly enabled by the use of USR, means that we can quickly search a much larger region of chemical space than previously possible. Fourth, the application of RF-Score, the first scoring function based on non-parametric machine learning, has been shown to result in more potent inhibitors than shape similarity alone, while maintaining an excellent hit rate. It is also encouraging that this initial version of RF-Score is already very competitive not only with established scoring functions, but with the consensus formed by all of them. These results are consistent with the observed superior performance in estimating binding affinity of diverse protein–ligand complexes using RF-Score [30,31] and more recently by other machine-learning-based scoring functions [41–43].

In practice, virtual screening methodologies are limited by the quality and availability of relevant experimental data (e.g. known active molecules and X-ray structures) as well as the degree to which their underlying assumptions align with the properties of the screened molecules and target protein (e.g. the scoring function performs well for that target). In the context of our study, there are a number of modifications that are likely to result in improved virtual screening protocols. First, research into more suitable intermolecular interaction descriptors and the exploitation of higher volumes of high-quality structural and interaction data should result in improved RF-Score predictability, as discussed in Ballester & Mitchell [30]. Also, because RF-Score identified different inhibitors than those from GOLD scoring functions, a consensus score based on all four scoring functions is likely to provide a better ability to identify binders, or at the very least a different set of new inhibitors. Furthermore, shape similarity and docking exclusively exploiting *M. tuberculosis* DHQase structures and inhibitors, instead of our mixed use of data for *S. coelicolor* and *H. pylori* DHQase, should allow better recognition of inhibitors of this version of the enzyme. On the other hand, Fischbach & Walsh [5] have argued that given current uncertainties as to how antibiotics get into bacterial cells and the structural diversity of antibacterial targets (and thus the molecules that bind them), libraries developed for other therapeutic areas may be just as likely to harbour hits as compound libraries developed for antibacterial screening. Consequently, we did not focus on the library design issue, although quantitative frameworks to design optimal HTS screening compound collections could be also beneficial for virtual screening performance in the future [44]. Most importantly, the extreme efficiency of USR permits one to exploit the chemical diversity contained in many billions of molecules in a time- and resource-efficient manner. Therefore, the use of much larger databases of molecules, each represented by a comprehensive ensemble of energy-accessible conformations, should lead to an even higher proportion of diverse inhibitors. Incidentally, we did not generate multiple conformers per molecule in our study, partly because the flexibility of the putative ligand in the binding site was going to be sampled during the pose generation stage. However, applying USR on a multi-conformer database instead would have led to the retrieval of more similarly shaped molecules that otherwise would have been missed.^4^ The application of such improvements is expected to result in additional DHQase inhibitors.

Overall, our results are directly relevant for those medicinal chemists who are interested in studying the selectivity of these new inhibitors with a view to using them as *in vivo* chemical probes [45]. These compounds could also be of interest as starting points towards generating lead compounds for rational drug design. These new inhibitors can now be evaluated for whole-cell antibacterial activity, and also undesirable properties such as hitting unwanted enzyme targets, to assess whether these are likely to be useful for developing leads. The hit-to-lead process is likely to require testing chemical modifications around each core scaffold, as none of the antibacterial hits found in intensive HTS campaigns were directly a lead [3]. However, both the number, and especially the variety of scaffolds of the hits maximizes the chances of at least some of the resulting series ultimately becoming a lead. We are disclosing the structures of all these new classes of DHQase inhibitors in the electronic supplementary material of this article to make such follow-up studies possible.

Finally, the reported methodology and proposed improvements can be applied to any other antibacterial target with resolved protein structures and known binders. This will be particularly advantageous for those targets for which no, or only structurally similar, active molecules are known. Surprisingly, despite the need for fast and cost-effective tools for hit identification, virtual screening is currently underused [46] and the antibacterial area is not an exception [6]. Virtual screening is, we believe, an excellent strategy for generating hits, though these may require significant experimental work to develop into useful leads. We hope that our study will contribute to encouraging budget-constrained academic laboratories to collaborate with virtual screening experts to find novel active scaffolds for the many validated molecular targets already available. Beyond antibacterials, validated virtual screening tools are also promising to tackle emerging antimicrobial drug resistance in other infectious diseases such as those caused by viruses and multicellular parasites (e.g. drug-resistant mutant influenza viruses [47] or resistance to existing antimalarial drugs [48]). Looking more broadly, the integration of virtual screening and HTS, which is still underdeveloped in any therapeutic area [34], can be extremely productive. For a new target with known substrate and structural model, virtual screening can be used to quickly identify potential chemical probes to investigate the target's druggability and *in vivo* response to dose-dependent modulation (both are often necessary to secure the funding for a full-scale HTS). For validated targets, virtual screening can help to greatly reduce the costs, increase the hit rate and speed up the operation of HTS [49] by enriching the screening library with a large set of molecules likely to be active. We believe that this practise would be very valuable in these targets where HTS performance has been unsatisfactory, as the latter may simply be due to the selected screening library containing few inhibitors in the first place. Lastly, a minority of targets are not amenable to HTS [50] due to problems associated with generating sufficient purified protein or substrate, or because the assay cannot be miniaturized to a robust HTS format. Such cases provide an obvious niche for the exclusive application of prospective virtual screening.

## Footnotes

1 *Streptomyces coelicolor* is a close non-pathogenic relative of *M. tuberculosis*, and thus is often used as an *in vivo* model for the characterisation of compounds that show promise *in vitro*.

2 Note that the same validation is not possible with any of the three GOLD scores, as these provide dimensionless units.

3 Hit means a chemically tractable, low micromolar inhibitor of the target and, where appropriate, at least 10-fold selectivity against the mammalian version of the target (none in the case of DHQase) [3].

4 A conformer registered in a database may be different from the bioactive conformation and thus multiple conformers per molecule are usually beneficial.
